# Editorial: Cognitive Hearing Mechanisms of Language Understanding: Short- and Long-Term Perspectives

**DOI:** 10.3389/fpsyg.2017.01060

**Published:** 2017-06-22

**Authors:** Rachel J. Ellis, Patrik Sörqvist, Adriana A. Zekveld, Jerker Rönnberg

**Affiliations:** ^1^Department of Behavioural Sciences and Learning, Linköping UniversityLinköping, Sweden; ^2^Linnaeus Centre HEAD, Swedish Institute for Disability Research, Linköping UniversityLinköping, Sweden; ^3^Department of Building, Energy and Environmental Engineering, University of GävleGävle, Sweden; ^4^Section Ear and Hearing, Department of Otolaryngology-Head and Neck Surgery and Amsterdam Public Health Research Institute, VU University Medical CenterAmsterdam, Netherlands

**Keywords:** cognitive hearing science, working memory, speech perception, language processing, hearing impairment

Cognitive hearing science is a relatively new field, which developed in response to an increasing awareness of the critical role of cognition in communication (Arlinger et al., 2009). Cognitive hearing science emphasizes the subtle balancing act between bottom-up and top-down aspects of language processing. Recent models of language understanding under adverse or distracting conditions have emphasized the complex interactions between working memory capacity, attention, executive functions, cognitive spare capacity and episodic and semantic long-term memory (Mishra et al., 2013; Rönnberg et al., 2013). This kind of approach has promoted a more comprehensive grasp of the interplay between bottom-up and top-down processes, including both online processes and long-term changes (positive or negative) relating to hearing impairment/deafness and aging.

The goal of this research topic (Cognitive hearing mechanisms of language understanding: Short- and long-term perspectives) was to encourage submissions that could push the field forward by suggesting behavioral and neural mechanisms that are important for online language processing, and for long-term cognitive change. Each of the 34 papers that are included in this research topic have contributed toward meeting this goal, and to furthering our understanding of the complex interplay between cognition and language. In addition to papers reporting original research, the research topic also includes both review and opinion and theory articles, giving us not only new empirical evidence, but novel approaches and theories drawn from existing knowledge and data.

## Ageing, cognition, and language

Many of the papers included in this research topic focus on the impact of aging on cognition and language. Carroll et al. showed that age-related differences in lexical access efficiency modulates successful speech recognition in noise in listeners with normal hearing, even when vocabulary size is matched between younger and older listeners. Karawani et al. investigated auditory perceptual learning in older adults with and without hearing loss. While both groups of listeners showed significant improvements on the trained conditions (compared to untrained listeners), generalization to non-trained tasks was limited. Heinrich et al. compared behavioral and self-report measures of aided speech perception in older adults with mild-to-moderate hearing loss. The results suggest that behavioral and self-report measures correlate more highly when they relate to similar speech situations; and that only behavioral speech perception measures correlate with cognition. Two articles focussed on sentence comprehension in older adults; DeCaro et al. report that once hearing acuity and working memory capacity have been accounted for, age does not significantly predict comprehension accuracy. Amichetti et al. were interested in whether aging and/or hearing loss affected the extent to which listeners relied on formal syntax vs. plausibility to successfully comprehend sentences. Like DeCaro et al., Amichetti et al. also found that (in all conditions but one) age did not significantly predict comprehension once hearing acuity and working memory capacity had been accounted for, but that age and hearing acuity may affect which comprehension strategy is used. Meister et al. investigated the effects of cognitive load on speech recognition with one or two competing talkers in older listeners with and without hearing loss. The results showed that listeners with hearing loss performed particularly poorly, and demonstrated a different pattern of errors, in conditions with two (compared to one) competing target talkers. These differences are attributed to impaired object formation and an increased demand on working memory.

## Translational research and methodological evaluation

In their review article, Souza et al. investigate the relation between hearing aid processing and working memory, concluding that evidence for a link between memory and wide-dynamic range compression is strong, yet further research is needed to investigate the links between working memory and other hearing aid processing strategies. Pisoni et al. also highlight the need for more research, in this case into the cognitive factors predicting speech and language outcomes in cochlear implant users, suggesting that research into basic domain-general learning abilities is particularly lacking.

Moulin and Richard investigated the role of context and lexical factors on a standard clinical spondaic word recognition in quiet task in 160 adult listeners with hearing loss. Their results indicate that the use of context decreased with hearing loss once the pure tone average exceeded 55 dB HL, and that there is a significant age effect on the relation between word recognition and word frequency. Koch et al. were also interested in the clinical and real-world utility of the speech materials used in their study of acceptable noise level test outcomes. They found that acceptable noise levels were correlated with self-reported hearing problems, and that the repeatability of the acceptable noise level test was not affected by the use of more natural speech materials. Frtusova and Phillips also focussed on the impact of methodological choices on test outcomes, showing that older adults obtained benefit to performance in a working memory task when stimuli were audiovisual as opposed to audio-only. Both behavioral and electrophysiological measures indicated that this benefit was more pronounced for listeners with poorer hearing than for those with better hearing.

## Bilingualism, signed-, and native- and non-native language

A number of papers in the research topic focussed on native and non-native language use, or bilingualism and its relation to cognition. Schneider et al. compared the short-term memory performance of older and younger native English speakers to a group of younger people for whom English was a second language. The results showed that older adults had poorer memory scores than younger adults, but that there was no difference in performance between the younger adults for whom English was a native language, and those for whom English was a second language. Schmidtke investigated the speech understanding in noise scores of monolingual English and Spanish-English bilingual young adults. The results indicated that speech understanding in noise improves with greater language exposure and that working memory does not provide additional predictive power, likely due to the large amount of variance shared between working memory and language proficiency. In their review, Calvo et al. argue that while many previous studies have observed that bilingualism does not affect working memory, certain aspects of working memory may be enhanced by bilingualism, yet methodological choices often mean that such improvements are difficult to observe.

Holmer et al. also investigated the effects of native and non-native language, but in signed rather than spoken language. The results are used as support for a developmental version of the Ease of Language understanding model, the D-ELU, which is outlined in the article. Rudner et al. also focussed on sign language by investigating the effects of load and distinctness on performance on a sign-based memory task by young adult listeners with no previous experience of sign language. The results showed that working memory load increased when sign distinctness decreased, providing support for an amodal mechanism active even when a pre-existing semantic representation is missing.

## Working memory and cognition in young normally-hearing listeners

Füllgrabe and Rosen caution against assuming that working memory has the same importance to speech-in-noise processing in young adults with normal hearing as it does in older adults with hearing loss. The results of their meta-analysis suggest that variations in working memory capacity account for only approximately 2% of speech-in-noise recognition scores in younger adults with normal hearing. In contrast, Hadar et al. report that working memory is of importance for speech processing in younger adults with normal hearing, using processing time as opposed to accuracy as an outcome measure in their eye-tracking study.

In relation to the interplay between communication, working memory and cognition, Marsh et al. (see also corrigendum) report results from a study showing that background noise interferes with gist processing of spoken messages. Finally, Beaman and Jones discuss mechanisms involved in forgetting in short-term memory, focussing on different forms of overwriting.

## Communication-related disorders

A number of articles in this research topic report results pertaining to individuals with communication-related disorders other than hearing loss. Murphy et al. looked at phonological and non-linguistic auditory training on the phonological skills of children with speech sound disorder. While neither training condition led to improvements in phonological skills, the non-linguistic auditory programme did lead to improvements in both auditory and cognitive measures. Söderlund and Jobs investigated differences in speech recognition thresholds in children with and without attention deficit hyperactivity disorder when exposed to noise. The findings indicated that children with attention deficit hyperactivity disorder had higher speech recognition thresholds than a control group, and that this difference disappeared when the children were exposed to white noise. Frölander et al. investigated theory of mind and executive function in adults with Alström syndrome (a genetic disorder associated with a variety of symptoms including vision and hearing loss), finding that the group with Alström syndrome performed significantly poorer than a control group in both types of task. Usher syndrome is another genetic disorder associated with hearing and vision loss, and was the focus of research reported by Henricson et al. The findings showed that adults with Usher syndrome performed more poorly than a control group on tests of phonological processing, and on tests involving fast visual or phonological processing. Henricson et al. suggest that these difficulties may contribute to explaining the high levels of fatigue often reported by individuals with Usher syndrome.

## Listening effort

Listening effort was the subject of a number of papers in the research topic. Wagner et al. used pupillometry and response times to show that lexical competition is associated with effort, and that degraded speech affects the timing of information processing, leading to increased effort. McMahon et al. also used pupillometry, along with alpha power, to investigate listening effort. The findings showed that these two measures show similar trends when participants processed highly intelligible speech, but seemed to diverge with degraded speech. Wendt et al. were also interested in comparing different measures of listening effort, finding that pupillometry and subjective rating scales index different aspects of effort, and that the syntactic complexity of speech can affect effort even when intelligibility is high. Francis et al. also report differences in subjective and physiological measures of listening effort, finding that masking affects physiological measures to a greater extent than subjective measures of listening effort.

## Neurophysiology

In Cardin's review of the effects of aging and hearing loss on cortical auditory regions, parallels are drawn between the cortical mechanisms that are engaged when young listeners with normal hearing engage in effortful listening, and those that are engaged in all listening for older listeners and those with hearing loss. Marsh and Campbell were also interested in the neurophysiological mechanisms underlying auditory processing. Their article introduces the new early filter model which suggests that complex sounds are processed early on by a subcortical filter under cholinergic top-down control.

In addition to the themes outlined above, a number of other topics were the focus of articles included in this research topic. Kishida et al. reported work on the synthesis of intelligible noise-vocoded Japanese speech. Ruigendijk and Friedmann investigated the effects of hearing impairment on the comprehension and repetition of movement-derived sentences in German-speaking children. Heald et al. introduce a new framework for perceptual plasticity relating to auditory object recognition, focussing on its context-dependent nature. Skoog Waller et al. investigated estimates of a speaker's age, finding that speakers were estimated as having a younger age when the speech rate was fast, and an older age when the speech rate was slow.

Taken together, the manuscripts included in this research topic represent important advances in the field of cognitive hearing science. Specifically, the papers demonstrate evidence of the involvement of cognition at all levels of speech perception, from basic word recognition through to comprehension. The variety of outcome measures included has enabled us to gain further insight into the impact of methodological choices on the likelihood of observing an effect of cognition, and indeed how the effect of these choices may vary depending on the participant group being tested. Further evidence is provided of the negative impact of hearing loss and aging on communication, and the role of cognition in ameliorating these negative effects. An important contribution of this topic is finding evidence for the importance of cognition on communication not just for older listeners or those with hearing loss, but also for listeners with a variety of communicative impairments, and indeed young listeners without hearing loss (albeit dependent on the outcome measures employed). We also see a lot of evidence for the moderating role of linguistic factors (e.g., native/non-native language, syntactic complexity) on the relation between cognition and communication.

Together these results provide support for five of the six predictions based on the ease of language understanding (ELU, see for example Rönnberg et al., 2013) model outlined by Rönnberg and Rudner (under review), namely that the effect of signal distortion, early attention mechanisms, use of phonological and semantic cues, and effort are all linked to working memory, and that working memory also affects the perception of sign language. The only prediction that is not addressed here is that there will be differing effects of hearing loss on long-term memory, but not on working memory.

We hope that this research topic will help to inspire many more studies in the field of cognitive hearing science. This area will continue to advance and contribute to both our understanding of the mechanisms underlying language understanding, and to improving assessment and treatment options for people with a communicative impairment.

## Author contributions

All authors listed, have made substantial, direct and intellectual contribution to the work, and approved it for publication.

### Conflict of interest statement

The authors declare that the research was conducted in the absence of any commercial or financial relationships that could be construed as a potential conflict of interest.
